# Glycemic Control in Patients with Diabetes on Peritoneal Dialysis: From Glucose Sparing Approach to Glucose Monitoring

**DOI:** 10.3390/life15050798

**Published:** 2025-05-17

**Authors:** Aleksandra Kezić, Selena Gajić, Ana Račić Ostojić, Ivana Bekić, Ana Bontić, Jelena Pavlović, Marko Baralić, Ljiljana Popović

**Affiliations:** 1 Clinic of Nephrology, University Clinical Center of Serbia, Pasterova 2, 11000 Belgrade, Serbia; selenagajic@yahoo.com (S.G.); ana.bontic@med.bg.ac.rs (A.B.); jelena.pavlovic@med.bg.ac.rs (J.P.); marko.baralic@med.bg.ac.rs (M.B.); 2 Faculty of Medicine, University of Belgrade, Dr. Subotića 8, 11000 Belgrade, Serbia; ljpopovic@beotel.net; 3 Department of Nephrology, Clinical Hospital Center Zemun, Vukova 9, 11080 Belgrade, Serbia; aostojic7@yahoo.co.uk; 4 Children’s Hospital for Lung Diseases and Tuberculosis, Clinical Hospital Center “Dr Dragiša Mišović-Dedinje”, Heroja Milana Tepića 1, 11000 Belgrade, Serbia; ivana.bekic@dragisamisovic.bg.ac.rs; 5 Center for Diabetes and Lipid Disorders, Clinic for Endocrinology, Diabetes and Metabolic Diseases, University Clinical Center of Serbia, Dr. Subotića 13, 11000 Belgrade, Serbia

**Keywords:** diabetes mellitus, peritoneal dialysis, icodextrin, continuous glucose monitoring

## Abstract

Optimized glycemic management is crucial for controlling atherosclerosis and consequent cardiovascular morbidity in patients with diabetes. Due to the continuous glucose burden from glucose-containing peritoneal dialysis (PD) solutions, PD patients with diabetes experience difficulties in glucose level regulation with glucose hypervariability and worsening dyslipidemia. Even in non-diabetic PD patients, glucose-containing PD solutions aggravate insulin resistance and cause overweight. Additionally, glucose degradation products (GDP) from glucose-based PD solutions provoke oxidative stress and complex inflammatory processes, leading to chronic deleterious and fibrotic peritoneal membrane changes. In this narrative review, we searched the literature using PubMed, MEDLINE, and Google Scholar over the last three decades to summarize the most important facts relevant to the presented issues, aiming to inform both endocrinologists and nephrologists in providing the best currently available care for people with diabetes on PD. We not only focus on adequate tailoring of insulin therapy adapted at the time of PD exchange with hypertonic glucose solution., but also emphasize the use of continuous glucose monitoring (CGM) that allows assessment of mean glucose values and time spent in normal, hypo, and hyperglycemia. However, the routine use of CGM in PD patients is limited due to high cost, and hemoglobin A1c (HbA1c) analysis is still recommended as a basic clinical tool for the assessment of glycemic control. Possible choices of antidiabetic drugs were considered given the narrowed choice due to contraindications for metformin and sulfonylurea. The other important therapeutic approach in PD patients with diabetes is using glucose-sparing PD regimens based on icodextrin and amino acid PD solutions with the addition of just one or two bags of low glucose concentration PD solution daily. This glucose-sparing approach not only reduces the glucose load and improves glycoregulation with correction of the lipid profile but also maintains the viability of the peritoneal membrane by reducing the harmful effects of GDPs.

## 1. Introduction

Diabetes mellitus (DM) is not just the most prevalent cause of end-stage renal disease (ESRD) but carries a risk for worse survival compared to non-diabetic patients with ESRD [1,2,3]. Peritoneal dialysis (PD) accounts for about 11% of all patients undergoing chronic dialysis treatment [4]. Regardless of dialysis treatment, the leading cause of death in DM patients is a cardiovascular event, including myocardial infarction, heart failure, and cerebrovascular disease [5]. Generally, potential risk factors for patients on peritoneal dialysis (PD) are glucose burden and hyperinsulinemia with dysregulated metabolic response and consequent dyslipidemia and central obesity [6]. This issue is even more pronounced in patients with Type 2 diabetes (T2D). Considering chronic hyperglycemia as the most important causative factor for all micro and macrovascular complications that underlie cardiovascular morbidity and consequent mortality in patients with DM, it can be assumed that glycemia correction and monitoring in these patients who are on PD are more challenging tasks than in the predialysis period for both nephrologists and endocrinologists. Additionally, chronic use of conventional glucose-based PD solutions alter anatomical integrity by inducing fibrosis of peritoneal membrane [7]. Therefore, we aim to summarize the current knowledge on the impact of glucose-based PD solutions on glycemia and consequent metabolic disarrangements and their potential association with survival and evaluate therapy approaches and strategies for optimizing glycemic management in patients with DM undergoing PD, emphasizing the clinical role of non-glucose-based PD solutions and the possibility of continuous glucose monitoring (CGM).

## 2. Literature Search Strategy

We conducted a comprehensive search for the literature published in English in the last thirty years in PubMed, MEDLINE, and Google Scholar. The terms that were used in the search alone or in combination were “peritoneal dialysis, “diabetes mellitus”, “glucose-based peritoneal dialysis solution”, “icodextrin”, “insulin therapy”, “glucose monitoring”, “continuous glucose monitoring”, “dyslipidemia”, “insulin resistance”, “amino acid-based peritoneal dialysis solution”, “antidiabetic drugs in peritoneal dialysis”, “mortality in peritoneal dialysis”. The list of the identified relevant studies was scrutinized for additional citations. For further analysis, we considered prospective randomized and observational studies, cross-sectional studies, case–control studies, case reports, systematic reviews, and meta-analyses related to glucose management and associated metabolic complications in PD patients with DM. Our search analysis resulted in 2354 records, but after removing duplicates, 1825 remained. After the exclusion of 1511 articles not strictly related to the scope of this review, 314 abstracts were assessed for eligibility, and 151 full-text articles were included in the review (Supplementary Figure S1).

## 3. Survival of Diabetic Patients on PD

Many studies have investigated dialysis patients’ survival, depending on dialysis modality, follow-up period, and the presence or absence of DM. All studies indicate the severity of the present cardiovascular comorbidities, which has the most significant impact on the outcome of the treatment of dialysis patients, whether they are on hemodialysis (HD) or PD. Although DM was associated with worse survival, irrespective of dialysis mode of treatment PD or HD, recently, a tendency for mortality decrease in dialysis-dependent patients with diabetes was noticed [2,8]. There seems to be a time-dependent trend in the relative risk of death, pointing to PD as a dialysis method associated with better or equal survival during the first two years of dialysis [9].

In the group of more than 20,000 incident dialysis patients, PD patients had 48% lower mortality compared with HD patients over the first 2 years of dialysis therapy, independent of modality switches or differential transplantation rates [10]. In the first few years of dialysis treatment, it was observed that persons without diabetes on PD had the best survival, followed by the same survival of PD persons with diabetes and HD persons without diabetes, while persons with diabetes on HD had the worst survival [11]. However, additional analysis showed that older patients with diabetes did not benefit from PD; on the contrary, an increased risk for mortality was even observed in this population. Similar results were confirmed by authors of a Dutch study, which showed that during dialysis, the advantage of PD compared to HD in survival decreases [12]. A possible explanation for the worse survival of older persons with diabetes on PD might be a greater incidence of infections, namely PD-associated infections such as peritonitis [9,13]. Also, selection bias may influence results as patients with diabetes and heart failure with hemodynamic instability are selected for PD, but this combination of comorbidities carries a high risk for mortality. A recent meta-analysis confirmed the increased mortality risk for PD versus HD in older patients with DM or comorbidities who have been on dialysis treatment for more than 3 years [14]. Propensity-matched comparison of mortality between PD and HD patients with T2D showed no difference in mortality irrespective of whether there are the first 2 years or over the 2 years of dialysis treatment, but different mortality predictor patterns were observed [15]. At the initiation of PD treatment, advanced age and increased cholesterol were independent risk factors associated with mortality, whereas, for HD patients, lower body mass index (BMI) and higher HbA1c were associated with higher mortality. One retrospective cohort study showed that HD and PD were not significantly different regarding patient survival, but subgroup analysis favored PD as a method of choice in patients with diabetes [16]. Meta-analysis of 17 cohort studies, which included 113,578 incident dialysis patients, indicated that PD and in-center HD carry similar survival rates [17]. The interaction term for diabetes and dialysis modality was non-significant.

If the patients are eligible for both modalities of treatment, HD or PD, similar mortality was recorded among incident dialysis patients in both groups, and this effect of dialysis modality on survival does not appear to change over time [18]. PD is undoubtedly valuable as an initial method of treatment for ESRD patients and can also be a long-term choice of treatment for younger persons with diabetes, but in older patients, in order to achieve a long-term, effective PD method, there is a need for timely treatment, i.e., mitigating the risk factors associated with the PD method per se to improve the survival in this group of patients. It was concluded that a higher mortality rate in patients with diabetes on PD was associated with cardiovascular disease, protein–energy wasting, and low residual renal function (RRF) [19]. One of the most important causes of increasing mortality in PD patients associated with more extended PD use is the detrimental, cumulative effects of glucose degradation products (GDP) formed in the process of heat sterilization of PD solutions [20]. GDPs strongly react to form advanced glycation end-products, which are a known cause of arteriosclerosis and consequent numerous microvascular complications [21]. Additionally, the most important PD risks in patients with diabetes are dysregulated metabolic response to glucose with consequent hyperinsulinemia/insulin resistance, dyslipidemia, and accelerated atherosclerosis.

## 4. Metabolic Risk Factors in Patients with Diabetes on PD

### 4.1. PD Solutions Related Factors

One of the main problems for metabolic dysregulation in persons with diabetes treated by PD is the continuous glucose burden from glucose/containing PD solutions. It can reach quantities equal to a daily intake of 100 to 300 g of glucose, which can cause hyperglycemia in a substantial number of PD patients, even for those who do not have DM [22]. The association between the concentration of glucose in the PD solution and daily mean blood glucose concentration recorded by CGM was demonstrated [23]. Actually, persons without diabetes on PD are never truly fasting. Systemic glucose absorption is a constant stimulus for insulin secretion, which aggravates insulin resistance (IR), and consequent micro- and macro-vascular atherosclerotic changes lead to increased cardiovascular risk for patients undergoing PD [24]. Continuous glucose burden from PD dwells results in more glucose uptake, leading to high blood glucose levels, increased IR, dyslipidemia, overweight, hypertension, and even new-onset DM (NODM) in previously non-diabetic PD patients. IR plays an important role in the development of cardiovascular diseases in both patients with and/or without diabetes [25]. IR is predictive of cardiovascular disorders and is a common finding in uremia [26,27,28]. Recently, many researchers have focused on the application of plant polysaccharides to reduce IR and treat T2DM and other metabolic diseases [29]. In patients initiating PD, triglyceride/glucose index (Ty/G), which is a marker of IR and metabolic syndrome (MS), predicted cardiovascular mortality [30]. This MS impacts mortality, especially cardiovascular mortality [6,31]. Even more, PD patients seem to have an increased cardiovascular mortality risk associated with MS compared to HD patients [32]. Additionally, patients undergoing PD with poor glycemic control have a higher risk of death [33]. A direct relationship between the amount of glucose in the dialysis fluid and the survival of PD patients has been demonstrated. The higher peritoneal dialysate glucose content independently predicted all-cause mortality and was an independent risk factor for cardiovascular mortality [34,35].

Besides hyperglycemia, already mentioned GDP, accumulated in the dialysis solution during heat sterilization, are rapidly absorbed from the peritoneal cavity. These substances further undergo non-enzymatic chemical transformation into advanced glycation end-products (AGEs) that contribute to the development of atherosclerosis. The interaction of AGE with cell surface receptors for AGE (RAGE) results in increased oxidative stress in numerous cells and increased RAGE expression. As a result of these interactions, inflammatory, thrombotic, and fibrotic reactions take part in the formation of atherosclerotic lesions. The same pathophysiological process is involved in the development and progression of atherosclerotic cardiovascular disease in patients with diabetes, for which cumulative hyperglycemic exposure plays a central role [36,37,38]. It has been shown that circulating AGE levels predict total and cardiovascular disease mortality in Type 1 diabetes (T1D) and T2D patients [39,40]. In addition to atherosclerosis, GDP and AGE lead to detrimental effects on the peritoneal membrane, causing structural and functional changes manifested as ultrafiltration failure and even the development of peritoneal sclerosis [41,42]. The first step in that process is the apoptosis of peritoneal mesothelial cells and endothelial cells induced by GDPs, which leads to the epithelial–mesenchymal transition of peritoneal mesothelial cells [7]. In this complex process of chronic inflammation that leads to peritoneal fibrosis, except for GDPs, gut microbiota alterations are recently becoming an emerging area of interest [43,44]. Future studies must elucidate the extent to which the peritoneal dialysis process and the specific effect of GDP play a role in the development of gastrointestinal dysbiosis.

### 4.2. Dyslipidemia in PD Patients

One of the metabolic risk factors implicated in atherosclerosis and peritoneal membrane chronic injury is dyslipidemia, which is a feature of chronic kidney disease (CKD) independently of diabetes [45]. The interplay of uremia-induced oxidative stress, inflammation, and IR induces specific lipid abnormalities during CKD. PD significantly amplifies these effects and exacerbates dyslipidemia and IR by continuously exposing the peritoneal membrane to glucose-based PD solutions and the loss of proteins in the dialysate [24,46,47]. Except for increased hepatic lipogenesis induced by IR and enhanced very-low-density lipoprotein (VLDL) cholesterol production, high-density lipoprotein (HDL) formation is decreased due to peritoneal protein loss, which is involved in that process [45]. In diabetes, regardless of PD, a lack of apolipoproteins (Apo) AI and apo AII and increased HDL clearance are the main reasons for low HDL [48]. Since low HDL levels are associated with infections and increased atherosclerosis with systemic inflammatory response, it is important to maintain adequate HDL levels in PD patients with diabetes. Indeed, patients with diabetes and low HDL levels were at higher risk for peritonitis compared to other PD patients [49]. Additionally, hepatic response to peritoneal protein loss is increased lipoprotein production, which exacerbates the dyslipidemic profile by increasing the levels of low-density lipoprotein (LDL) and VLDL. Altogether, these lipid changes in PD patients are similar to dyslipidemia in diabetes, indicating a significantly higher risk in patients with diabetes on PD, as well as the necessity of strict glycoregulation and treatment of dyslipidemia in this patient population.

## 5. New-Onset DM (NODM) in PD Patients

Otherwise, NODM occurs more frequently in dialysis patients than in the general population. If we talk about NODM as a part of the new-onset glycemic disturbance, which includes impaired glucose tolerance (IGT) and impaired fasting glucose (IFG), then around half of PD patients may develop a glucose impairment [50]. These are the results of a meta-analysis, which included nine studies involving 13,879 PD patients. The Global Fluid Study reported NODM in 3.7% of incident PD patients and 5.4% of prevalent PD patients [51]. However, NODM and DM are both diagnosed if fasting glucose ≥ 7.0 mmol/L or 2 h plasma glucose > 11.1 mmol/L in the oral glucose tolerance test (OGTT) with HbA1c threshold of ≥6.5% as the third criterion according to American Diabetes Association (ADA) [52]. In PD patients, fasting glucose (FG) seems inappropriate because of significant peritoneal glucose absorption during blood sampling. The same can be applied to the OGTT results. That is why different definitions of FG or criteria for NODM in PD patients exist in different studies [53]. However, fasting glucose ≥ 7.0 mmol/L as a criterion for DM can be found more often than fasting glucose ≥ 11.1 mmol/L [54,55,56,57]. Risk factors for NODM in dialysis patients were higher age, female sex, and cardiovascular disease [55,56,58]. Although it would be expected that there is a higher prevalence of NODM in PD patients than in HD patients due to continuous glucose load and absorption, some studies show that NODM is more common in HD patients [54,59]. On the contrary, the study conducted in Taiwan on 36,879 incident HD patients and 6382 incident PD patients from 2000 to 2010 confirmed that PD patients are at a higher risk of developing new-onset diabetes than HD patients [60]. These varying results of NODM rates in PD and HD populations are probably influenced by study design, PD prescription, and patient selection, including age and ethnicity [53]. It was shown that incidence of IR had been correlated with using 4.25% hypertonic dialysate [61]. Additionally, the use of icodextrin solution might reduce the risk for NODM in PD patients, but prospective studies are necessary to confirm it [60].

## 6. Glucose-Sparing PD Regimens

### 6.1. Biocompatibility of PD Solutions

The aforementioned data indicate the need and importance of reducing the total amount of glucose and exposure to glucose during PD, especially in patients with diabetes. The optimal dialysis solution in PD achieves adequate ultrafiltration, which is determined by the solution’s osmolarity and preserves the anatomical and physiological integrity of the peritoneal membrane. The viability of the peritoneal membrane is affected by the combined effects of glucose, GDP, and low pH. For the above reasons, efforts have been made in recent decades to produce a solution with a different osmotic agent, a neutral pH value, and the presence of cytoprotective agents. This would fulfill the biocompatibility criteria. Commercially present non-glucose-based PD solutions are icodextrin and 1.1% solution of amino acids. A low-glucose dialysis regime means replacing one dwell with a glucose-based dialysis solution by either icodextrin or amino acid-based dialysis solution, or even two dwells with glucose-based dialysis solution might be replaced by icodextrin and amino acid dialysis exchange.

### 6.2. The Role of Icodextrin in PD Regimen in Patients with Diabetes

Icodextrin is an iso-osmolar mixture of corn starch-derived high molecular weight glucose polymers containing lactate as a buffer. Due to very slow absorption from the peritoneal cavity, it maintains colloid osmotic pressure for a more extended period (up to 12–16 h), leading to increased ultrafiltration through small pores [62]. This sustained ultrafiltration during very long dwells is beneficial for anuric patients and even patients with RRF who are fast transporters and in whom adequate ultrafiltration without icodextrin can be achieved at the cost of increased exposure of peritoneum to high glucose concentration solutions. When two glucose-based exchanges were replaced with one icodextrin exchange, higher water and sodium removal with slightly lower urea and creatinine removal were achieved [63]. In non-diabetic patients, substituting glucose with icodextrin for the long dwell improved insulin IR measured by the homeostasis model assessment (HOMA) index [64].

Li and coworkers presented the results of two clinical trials (IMPENDIA and EDEN trials) that examined the systemic effects of glucose-sparing PD regimens in patients with diabetes. The glucose-sparing regimen was tailored using icodextrin and amino acid solutions instead of two bags of glucose-based solutions [65]. After a 6-month follow-up period, treatment with the glucose-sparing PD regimen improved metabolic indices such as significantly decreased glycosylated HbA1c value and decrease in plasma triglyceride, VLDL, and apo B level [65,66]. This decrease in plasma triglyceride, VLDL, and apo B levels induced by less PD glucose burden is a less atherogenic lipoprotein profile, and it is consistent with the known adverse effects of glucose on apo B metabolism [66]. It is important to emphasize that the percentage of patients on lipid-lowering medications at baseline was equivalent in each study group. Taking into consideration that a higher level of apo B was found in previous studies to be closely associated with cardiovascular risk, the reduced apo B level observed in this study in PD patients with diabetes is a promising finding [67,68].

The only negative result of the IMPENDIA/EDEN trials was an increased risk of extracellular fluid volume expansion. It seems contradictory considering the fact that the first indication for icodextrin use is volume overload since icodextrin produce sustained and increased ultrafiltration. However, if the remaining part of the PD regimen was tailored to use PD solutions of the lowest glucose concentration and this was not sufficient to achieve adequate ultrafiltration according to the present cardiovascular comorbidity, then observed volume expansion can be explained.

Although a more extended follow-up period may be required to fully evaluate survival and cardiovascular outcomes, and the changes in HDL cholesterol and LDL cholesterol were not noticed, the results of these trials are encouraging. However, other studies point to a tendency to increase HDL cholesterol and LDL cholesterol decrease after prolonged use of icodextrin [67,69]. Switching from glucose-containing dialysis solution to icodextrin in a prospective, multicenter open-labeled study led to an improved lipid profile expressed by decreased concentration of total and LDL cholesterol and triglycerides, particularly in patients with poor glycemic control [69]. Interestingly, there was no change in overall HbA1c levels compared to baseline values; however, in patients with baseline HbA1c ≥ 6.5%, a significant decrease in HbA1c was noticed. HDL cholesterol did not differ at any point during the study, but in patients with very low values of HDL cholesterol, a tendency to increase was observed [69]. Detailed analysis of lipoprotein fractions using high-performance gel permeation chromatography (HPGPC), which can separate lipoproteins into 20 fractions, e.g., particles of different sizes and densities, revealed that icodextrin may be effective for reducing cholesterol levels in small and very small LDL via a mechanism different from that for statins [70]. At the same time, multivariate analysis showed that icodextrin use was positively associated with cholesterol proportions in the very large HDL (*p* = 0.040), large HDL (*p* = 0.047), and medium HDL (*p* = 0.009) [70]. Interestingly, no significant differences in cholesterol levels determined as LDL and HDL were observed between the patients using icodextrin and patients treated only with glucose-based PD solutions. This study was conducted on 49 patients, of which 21 had diabetes. An observational study of hemodialysis patients showed that the smaller size of LDL particles is related to the poor prognosis of patients, not total cholesterol, LDL cholesterol, or HDL cholesterol levels [71]. The complex issue of HDL cholesterol is emphasized by finding that HDL cholesterol consists of diverse proteins and lipids and can be classified into different subclasses based on size and density, which can be changed dynamically in different diseases [72]. This means that HDL cholesterol level cannot be a reliable measure of cardioprotection, and some studies’ results must be interpreted with caution. Additionally, the meta-analysis comprising 13 eligible studies with 850 patients showed that icodextrin had advantages over the conventional glucose-based PD solution in correcting lipid profile and HDL-cholesterol level [73]. Decreased triglyceride levels and increased HDL cholesterol concentrations were more significant in long-term treatment with icodextrin, showing a time-dependent manner. All of the above beneficial effects of icodextrin administration are particularly desirable in patients with diabetes, in whom, besides volume control, correction of metabolic disarrangements is of equal importance (Table 1).

The icodextrin solution has other beneficial effects on patients with diabetes, such as improving technique survival [74,75]. The favorable effect on RRF and peritoneal membrane functions is questionable, e.g., different results were reported. In a follow-up after 12 months, daily urine volume decreased faster in the group of patients treated with glucose solutions than patients who used the icodextrin solution [76]. A systematic review of randomized controlled trials investigating icodextrin use showed no benefits for better preservation of RRF than glucose-containing solutions [77]. Of course, it is not always clear whether the prescription of a PD regime with the use of a higher amount of glucose is a risk factor for a worse outcome or whether hypervolemia is actually already present as a consequence of low RRF and inadequate ultrafiltration capacity of the peritoneum and is, in fact, the leading cause of increased cardiovascular morbidity [34]. In order to achieve euvolemic status, one must consider residual RRF and tailor the PD regimen to optimize ultrafiltration, for which the icodextrin solution plays an important role.

Regarding the transport characteristics, significant peritoneal membrane changes under icodextrin cannot be easily assessed since patients with faster transport status are preferentially chosen to be dialyzed using icodextrin. That is why deterioration in peritoneal solute transfer rate (PSTR) over time was not noticed in the group of patients who used icodextrin [78]. The results regarding investigating the association of icodextrin with survival are inconsistent. Numerous studies observed the survival benefits of icodextrin-containing PD over glucose-only PD [74,77,79]. However, prospective international Peritoneal Dialysis Outcomes and Practice Patterns Study (PDOPPS) data did not show superior patient outcomes using icodextrin instead of hypertonic glucose solution [80]. It appears that the use of icodextrin was targeted to patients with more comorbidities, including diabetes, who were supposed to have worse survival chances. In another meta-analysis, it was concluded that the effects of icodextrin on patient survival remain uncertain [81]. Interestingly, icodextrin significantly reduced the first peritonitis episode incidence in incident PD patients with diabetes [75]. Icodextrin might help restore an imbalance between the sympathetic and parasympathetic nervous systems in PD patients with diabetes [82]. This imbalance is an important risk factor for sudden cardiovascular death and arrhythmia. Additionally, icodextrin use is associated with a lower risk for new-onset atrial fibrillation. This protective effect was even greater in PD patients with diabetes [83].

Based on all facts, it can be concluded that PD populations, especially patients with diabetes, could greatly benefit from icodextrin treatment over a longer period of treatment, not just because of better extracellular volume control but also due to improved metabolic indices, although a longitudinal study is needed to reveal the time-dependent effect of icodextrin on lipid profile. The International Society for Peritoneal Dialysis (ISPD) suggests the use of icodextrin once daily as the long-dwell dialysis solution in patients with diabetes in order to achieve better glycemic control [84]. There is the intention for icodextrin use more frequently, i.e., twice daily or in combined, bimodal solutions with glucose [85,86]. In selected patients with fluid overload who are older and have pronounced cardiovascular comorbidity, the benefits from two bags of icodextrin daily are expected to be greater than the potential harms. However, in older patients initiating PD in an incremental regimen, a double icodextrin dose with one glucose-based exchange did not reduce the cumulative incidence of the composite primary outcome compared to a single icodextrin dose with two glucose-based exchanges, although the higher ultrafiltration was recorded in a group on double icodextrin dose [87]. The primary endpoint was the proportion of patients stopping three bags/day after nine months of follow-up due to the need for the use of more hypertonic glucose solutions, change in dialysis modality, or death. The safety profile was the same as with the once-daily administration of icodextrin. Nonetheless, waiting for additional analyses and the longer-term effects of administering a double dose of icodextrin is necessary.

Previously mentioned studies investigating different aspects of icodextrin showed well-established safety, although allergic rash and severe skin reactions can occur [88]. One multi-center study found a significantly higher rate of maculopapular eruptions in patients treated with icodextrin (4.6%), while in a group of patients who were treated using glucose-based PD solutions, no skin eruptions were noticed at all [89]. These complications are rare but require medical attention.

### 6.3. Amino Acid-Based Dialysis Solutions

The other commercially available nonglucose-based dialysis solution is a 1.1% solution of amino acids. Amino acid PD solution has a similar ultrafiltration potential to 1.36% glucose-based PD solution, and the original purpose of implementation of this PD solution in clinical practice was the improvement of nutrition indices in malnourished patients [90]. Although the pH of the amino acid solution is 5.5, it is considered biocompatible due to the absence of glucose and GDP. As mentioned, IMPENDIA and EDEN trials showed the positive role of amino acid PD solution in a low-glucose dialysis regimen in improving metabolic indices in patients with diabetes [65]. However, there is a lack of studies investigating the impact of amino acid dialysate on lipid status. After 3 years of use of amino acid dialysate, a sustained decrease in serum triglyceride level was detected [91]. In a 6-month trial, a single daily exchange of 1.1% amino acid dialysate showed a trend to decrease in serum triglyceride level, although nonsignificant, it did not influence total cholesterol, LDL cholesterol, and HDL cholesterol level [92]. Since the use of amino acid-based PD solutions was not associated with severe adverse effects, these PD solutions could take place in dialysis treatment not just in patients with malnutrition, but they might have wider use as a part of glucose-sparing PD regimens. Beneficial effects on peritoneal membrane integrity were shown after the use of an amino acid-based PD solution [93]. The potential effect of amino acid-based PD solutions is increased blood urea, which reflects nitrogen load and consequent nausea and vomiting [94].

### 6.4. The Clinical Perspective of Biocompatible PD Solutions

The idea of applying biocompatible solutions is not only to reduce the local harmful effect of glucose and GDP on the peritoneum and thereby preserve the functionality of the peritoneum for a more extended period but also to reduce the harmful systemic effect of glucose and GDP, i.e., AGE. Inconsistent conclusions regarding the influence of neutral pH, low GDP PD solutions, and better survival of PD patients were observed [81,95]. Solutions with an optimal pH value and eliminating GDP still have glucose as an osmotic agent. Their application showed a beneficial effect on preserving RRF and diuresis [96]. The preservation of RRF was progressively better with an increased follow-up duration [81]. That is why ISPD recommends neutral pH low GDP PD solutions to preserve RRF [84]. Studies confirm decreased serum C-reactive protein (CRP) values using neutral pH low GDP PD fluids [97,98]. With the preservation of RRF, this downregulation of chronic inflammation and decreased vascular effects could benefit survival [95]. Interestingly, in the balANZ trial, VLDL, triglyceride, and HDL serum levels were not modified by a biocompatible PD solution. In that study, none of these lipid parameters were predictive of composite cardiovascular events or mortality [99]. Unfortunately, the effects of neutral pH low GDP PD solutions on peritoneal membrane function, PD-related infections, and technique are unclear due to a lack of prospective studies with longer follow-up time [100]. More prospective randomized studies are needed to give precise answers regarding the potential causative role and mechanism of decreased cardiovascular morbidity and mortality in PD patients treated by low GDP solutions, regardless of the presence of diabetes.

A novel glucose-sparing approach might be using osmo-metabolic agents in the PD fluid. It is not just the reduction in intraperitoneal glucose exposure, but it is also an approach that provides both ultrafiltration and mitigation of underlying systemic negative metabolic effects caused by high glucose absorption and GDP [101]. Osmo-metabolic agents used alone or in combination, exhibit osmotic and metabolic effects. L-carnitine and xylitol are two representative examples of osmo-metabolic agents that were investigated in some clinical studies and well-tolerated by patients without harmful effects. L-carnitine plays a role in fatty acid metabolism, modulating mitochondrial levels of acetyl-CoA and liver glucose production. Clinical studies in continuous ambulatory peritoneal dialysis (CAPD) patients that investigated adding L-carnitine to PD solutions demonstrated efficacious ultrafiltration, preserved urine volume, and improved insulin sensitivity compared to glucose-based solutions [102].

## 7. Glucose Monitoring in PD Patients with Diabetes

### 7.1. Long-Term Glycemic Indicators

Achieving optimal glycoregulation and avoiding clinical inertia is paramount to reducing the risk of complications’ development and progression. It is also crucial to define treatment targets for each patient individually, considering the presence of other risk factors, complications, and comorbidities.

Optimal glycoregulation in patients with diabetes on PD without frequent hypo and/or hyper episodes is a challenging task. Choosing the most reliable method to estimate glycoregulation is probably even more important than the mode of diabetes treatment (oral antidiabetic drugs and/or insulin). It is essential to avoid high glucose variability, which could further complicate the management of these patients. The 2022 American Diabetes Association (ADA) Standards of Medical Care in Diabetes and Kidney Disease: Improving Global Outcomes (KDIGO) 2022 Clinical Practice for Diabetes Management in Chronic Kidney Disease each provided recommendations for the best practice management of these patients [103,104]. Unlike persons with diabetes who are not dialysis-dependent, in patients with diabetes who are on chronic PD treatment, HbA1c analysis as a tool for assessment of glycemic control has limitations in accuracy and precision due to many factors such as uremic environment, renal anemia, iron, and vitamin B12 deficiency, treatment with iron and erythropoiesis-stimulating agents (ESA), and other factors that affect red cell turnover [105,106]. Since HbA1c production takes time, any factors that affect reduced erythrocyte survival, such as a uremic environment or bleeding, will lead to falsely lower values. The use of ESAs might decrease HbA1c value due to the increased proportion of immature erythrocytes in peripheral blood, which are supposed to have lower glycated rates than mature erythrocytes [107]. Nevertheless, according to guidelines of the ISPD, HbA1c should be measured at least once every 3 months in PD patients with diabetes [84]. The recommended value of HbA1c is around 7% (53 mmol/mol) and may be up even to 8.5% (69 mmol/mol) in older persons with diabetes on PD.

The results of numerous studies regarding the association of HbA1c values and PD patients’ survival are inconsistent. HbA1c levels greater than 8% were confirmed as significant risk factors for increased mortality [32,108]. On the contrary, in a study involving more than 1000 prevalent patients with diabetes undergoing PD, no associations between HbA1c levels and 2-year mortality were recorded [109]. However, when glycated albumin (GA) was used as a measure of glycemic control, then GA > 20% appeared to be associated with decreased survival of patients with diabetes on PD [108]. Prospective studies with longer follow-up and choice of adequate indicator of glycemic control should address the question of whether the metabolic benefits of glucose-sparing regimens might reduce the cardiovascular risk of PD patients.

However, alternative glycemic indicators such as GA and fructosamine also have their limitations in patients with diabetes on PD. GA reflects recent glycemic control lasting for 3 weeks, but plasma GA level can be falsely low or high depending on albumin metabolism influenced by inflammation, oxidative and uremic environments, increased loss by peritoneal dialysis effluent, or reduced renal clearance [110,111,112]. It was proved in several studies, regardless of the mode of dialysis (HD or PD), that GA was a better glycemic indicator than HbA1c in patients with diabetes [113,114]. However, when the GA/HbA1c ratio was compared between HD and PD patients, higher values were found in HD patients, suggesting that GA measurements in PD patients might also underestimate blood glucose levels [114]. It seems that GA value may reflect glycemic control in patients with diabetes treated by PD, but GA concentration is consistently lower than the plasma glucose concentration in PD patients with diabetes compared to HD patients with diabetes [115]. Due to the shortened half-life of albumin in circulation, which is in substantial quantity lost in PD effluent, PD patients with diabetes exhibit low GA concentrations for their plasma glycemic level compared with non-PD patients with diabetes.

Additionally, the GA/HbA1c ratio was significantly associated with 3-year mortality in dialysis patients with diabetes, independent of glycemic control, anemia, and malnutrition [109]. The clinical importance of the GA/HbA1c ratio is that it represents the impact of glycemic variability on the survival of dialysis patients with diabetes. This ratio is easily obtained from a single blood test and is a promising marker for predicting survival, pointing to worse survival if GA/HbA1c ratio values are higher than the range between 3.0 and 3.3 [116].

Another alternative glycemic indicator is fructosamine, which is ketoamine formed by glycation not just of albumin only, but also of a broader spectrum of proteins [117]. Although HbA1c underestimated mean plasma glucose levels in patients with T2D on PD, it appeared that besides CGM, fructosamine accurately reflected glycemic status [118]. It was shown that glucose values recorded by the CGM system correlated significantly with albumin-corrected fructosamine [119]. However, fructosamine shows greater fluctuation than GA depending on abnormal albumin metabolism or increased albumin and protein loss. Fructosamine gained importance as a promising glycemic indicator in conditions characterized by rapid glycemic variation, which requires short-term monitoring, as seen in patients on corticosteroid therapy or changing insulin regimen or in patients with red blood disorders or CKD [120].

### 7.2. The Role of Continuous Glucose Monitoring in Assessing Glycemic Variability

Capillary blood glucose monitoring is the most used method for assessing day-to-day glycemic variability. However, adherence to self-blood glucose monitoring is poor because of the inconvenience of finger-pricking. Since GA, like HbA1c, may underestimate glycemic control in PD patients with diabetes, the question remains how to find precise glycemic indicators that would be more accurate for the assessment of glycemic control and variability. PD can lead to large fluctuations in glucose levels, causing hyperglycemia within minutes of starting peritoneal dialytic exchange. More precise glycemic variability assessment would adjust treatment changes, enhance self-adherence, reduce hypoglycemia anxiety, and improve the survival of these patients.

The answer could be CGM. Early implementation of CGM in PD patients with diabetes demonstrated for more extended daily periods blood glucose levels above recommended levels [121]. The latest KDIGO guidelines point to periodic use of CGM in addition to HbA1c in CKD, stages 4 and 5 [104]. The CGM system has revolutionized glucose monitoring and care of patients with diabetes over the past ten years. CGM allows continuous assessment of glycemic levels, providing mean sensor glucose as well as glucose variability and time spent in normal, hypo, and hyperglycemia during the day and night [122]. The ability to detect episodes of hypoglycemia is an important feature of CGM. The principle of CGM is based on the subcutaneous sensor’s measurement of interstitial glucose levels. The interstitial glycemia reflects plasma glycemia with a delay of several minutes, and measurements are taken every 5 to 15 min. A sensor and a transmitter are applied to the skin, and sensor data are transmitted to a receiver unit, where all the data can be viewed. Many exogenous/endogenous factors may affect sensor performance, such as hypoxia, uremia, pH, etc., but it remains to be clarified which factors disrupt the function of the sensors and to what extent. All available CGM models are not examined in dialysis patients and, thereby, not approved for use in this specific population [123].

Three types of enzymatic reactions (glucose oxidase (GOx), glucose dehydrogenase (GDH), and hexokinase) are currently being utilized in glucometers. GDH-based glucometers use three types of coenzymes, among which GDH with pyrroloquinolinequinone (GDH-PQQ) based systems should be avoided in PD patients using icodextrin because icodextrin is metabolized to maltose, which can cross-react with the GDH-PQQ-based glucometer system. As a result of falsely elevated blood glucose readings, excessive insulin treatment may lead to iatrogenic hypoglycemia. Commercial CGM systems based on GOx enzymatic reactions are not affected by icodextrin metabolites [124].

The most important CGM data is time in range, which includes the percent of time spent in the target range (TIR) (3.9–10 mmol/L) and the time below or above the target range. Time above range (TAR) reflects the percent of time spent above 10.0 mmol/L, and time below range (TBR) reflects the percent of time spent below 3.9 mmol/L with a Coefficient of variation less than 36% (%CV = SD-standard deviation of sensor glucose/mean sensor glucose). Since the CGM is a relatively new method still in development, there is relatively little data from case reports or studies with few patients investigating its role in improving glycemic control in persons with diabetes on PD (Table 2). It was reported that in three patients with almost the same values of HbA1c, different glucose profiles were measured by CGM [125]. Early observational studies using CGM showed hyperglycemia in a large proportion of time in PD patients [126]. While refilling with glucose-based dialysate, within 60 min, an increase in sensor glucose was detected; on the contrary, during icodextrin refilling, reduced sensor glucose was detected [119]. The other study conducted on both T1D and T2D patients on PD, in whom a mean HbA1c of 5.9% was detected, showed that 33% of the time, glucose level was above 10 mmol/L [108]. However, the use of the CGM system in 60 PD patients with diabetes detected the occurrence of hypoglycemia even in patients with HbA1c > 9% [127]. Switching the PD regimen from three 1.36% glucose exchanges and one 3.86% glucose exchange to two 1.36% glucose exchanges with one amino acid exchange and one icodextrin exchange was associated with lower glycemic variability [128]. Besides dialysate glucose concentration, glycemic patterns in PD patients are influenced by peritoneal membrane transport status. Mean sensor glucose and mean changes in sensor glucose after dialysate exchange in patients with diabetes were significantly greater in patients with high peritoneal transport versus in patients with high-average peritoneal transport [23].

The accuracy of newer models in patients on PD is still not known and not thoroughly assessed. Not all models for CGM are approved by their manufacturers for use in PD patients, because they were not tested in controlled studies in larger group of patients. The study which evaluated the accuracy and performance of a contemporary real-time CGM in PD patients with T2D showed satisfactory performance of this CGM. Medtronic Guardian Sensor 3 was reliable and accurate across a wide range of glucose levels [129]. The accuracy of the sensor was not influenced by acidosis, urea concentration, and volume overload. A small study amongst patients on CAPD, recently published, concluded that CGM demonstrated good accuracy with minimal impact from body composition or anemia [130]. For the general diabetes population, CGM is usually used in T1D, but its use in T2D has been less thoroughly investigated. In randomized trial on patients with T2D treated with basal insulin, CGM use after 8 months was associated with improved time in range [131]. CGM was even investigated to compare different glucose-lowering drug regimens [132]. All these presented results encourage the use of CGM regularly in a larger group of patients, including patients with T2D on PD. According to current guidelines for CGM in Dialysis Population, these people belong to a high-risk group, and it is recommended that they should spend more than 50% of the time, e.g., more than 12 h within the target range (3.9–10.0 mmol/L), less than 1% of the time below 3.9 mmol/L (less than 15 min per day) and less than 12 h per day above the 10.0 mmol/L [133]. The use of CGM systems in PD patients with diabetes might be of great help in adjusting insulin treatment to glucose influx from glucose-containing PD solution, thereby maintaining stable blood glucose without episodes of hypoglycemia. However, there are some limitations in using CGM for patients under dialysis, reflected in false hypoglycemic alerts requiring confirmatory blood glucose measurement [112]. Although there are other limitations, such as higher cost and lack of data from large studies investigating the benefits of CGM technology, previously mentioned studies suggest that CGM can be beneficial in people with diabetes on PD primarily to minimize glycemic variability. Also, there are some practical challenges of routine sensor insertion and removal that can limit the clinical implementation of CGM. Whether CGM can be used in a periodic manner with the same efficacy in detecting glycemic variability and hypoglycemia and improvement of glycemic control as when used every day needs to be elucidated. The summary of practical points regarding CGM use in PD patients with DM is presented in Table 3.

## 8. Treatment of Patients with Diabetes on PD

Antidiabetic therapy in patients with diabetes on PD must be adjusted according to current recommendations. As in patients without renal insufficiency, in patients on dialysis, the goals of DM therapy are the same: adequately controlled glycemia while avoiding hypoglycemia. In PD patients with diabetes, euglycemia should be maintained during PD changes, and episodes of postprandial hyperglycemia should be prevented. First of all, less stringent HbA1c targets are needed and should be individualized and maintained in the range between 7% and 8%.

Dietary advice should be adjusted to the recommended energy requirement for patients on PD and to a minimum dietary protein intake of 1.0 to 1.2 g/kg/day, aiming to maintain a good nutritional status and accounting for protein losses during PD exchanges [134]. The important issues are energy requirement and carbohydrate intake since PD solutions provide a certain amount of calories.

Among oral antidiabetics, the use of sulfonylureas and metformin is contraindicated, while the use of glucagon-like peptide receptor agonists (GLP-1 RA) is not recommended [135]. Although long-acting GLP-1 RA is recommended for patients with T2D and CKD who have not achieved glycemic targets despite using metformin and Sodium-glucose Cotransporter-2 Inhibitors (SGLT2i), experience in patients requiring dialysis is lacking [136]. However, pharmacokinetic studies showed no changes in efficacy in patients on PD and no additional serious side effects [137,138]. Treatment options include dipeptidyl peptidase-4 (DPP-4) inhibitors and pioglitazone. A reduced dose of sitagliptin at 25 mg and pioglitazone (15 or 30 mg) can be used [139]. Compared to DPP-4 inhibitors, pioglitazone is associated with lower all-cause mortality and major adverse cardiac cerebrovascular events, making these effects more profound in patients with dyslipidemia [140]. However, pioglitazone is less preferred because of known adverse effects such as fluid retention and macular edema [141].

SGLT2i are not approved for use in patients with diabetes on PD, although some studies favor their use in PD patients, but with different goals. The assumption was that SGLT2i can inhibit peritoneal glucose uptake, given that SGLT2 is expressed in the peritoneum and that SGLT2i reduces renal tubular glucose uptake. So, the main question is whether SGLT2i can inhibit peritoneal glucose uptake to maintain osmolality and increase ultrafiltration. Absorption of glucose from the peritoneal cavity upregulates the glucose transporter-1 (GLUT-1) gene and many profibrotic genes (e.g., TGF-β, VEGF, and CTGF) that stimulate the consequent development of peritoneal fibrosis [142]. GLUT-1 and SGLT2 receptors are expressed in peritoneal mesothelial cells [143]. In one experimental study, Canagliflozin inhibited HIF-1α/TGF-β/p-Smad3 signaling pathway, leading to a reduction in GLUTs and SGLT2 expression and peritoneal angiogenesis and fibrosis, which ultimately resulted in a decrease in glucose uptake and improved peritoneal transportation and ultrafiltration [144].

In an average of 31 months follow-up, PD patients with diabetes on SGLT-2i therapy had higher ultrafiltration [145]. The overall survival and the chance of urinary tract infection were not different between patients with and without SGLT2i. In patients receiving maintenance PD, the use of SGLT2i appeared to be associated with preserved RRF and improved blood pressure control, with the acceptable already-known safety profiles observed in patients with CKD who are not on dialysis [146]. These positive results obtained in recent human case series should be confirmed in randomized trials conducted on more patients, specifically aiming to clarify mechanisms of action on solute transport across the peritoneal membrane and RRF. In a study conducted on 50 patients with T2DM on insulin who were on automated PD, the results of dapagliflozin therapy were a decrease in insulin requirement and mean systolic blood pressure by 14 mmHg, while urine volume was increased by 253.2 mL/24 h and ultrafiltration volume was increased by 229.9 mL [147].

For patients with diabetes on insulin therapy, a basal-bolus regimen is primarily recommended, consisting of four daily doses (bolus insulin before meals with basal long-acting insulin in the evening). There is a lack of evidence to implement recommendations on specific insulin dose adjustments according to the additional impact on glycemia due to peritoneally absorbed glucose [148]. However, the dosage of insulin increases at the same time with the introduction of a 2.5% glucose PD solution [149]. That is why titration and insulin dosage must be individually adapted to each patient, and the target glycemia values must be according to the existing complications and comorbidities and the total cardiovascular risk [128]. Multiple-daily-injection of 0.5 units of insulin per kilogram using 50% of the dose as basal insulin and the other 50% of insulin dose divided into three premeal bolus doses, achieved fasting glucose target between 5.5 and 7.2 mmol/L (100–130 mg/dL) and 2 h post-meal glucose levels lower than 10 mmol/L (<180 mg/dL) [150]. The dose of basal insulin was titrated every 3 days. In some patients whose glucose excursions with PD are greater than with food (meal) intake, a premixed insulin regime such as 70/30 insulin can be suitable [139]. In patients with diabetes who use an insulin pump, increasing the basal rate during PD (especially in patients on automated peritoneal dialysis) and correcting the insulin-to-carbohydrate ratio to avoid postprandial hyperglycemia is recommended. They should also correct insulin sensitivity factors to prevent hypoglycemic episodes. The key is to avoid hypoglycemic episodes and high glucose variability, which can be devastating for these patients and may contribute to the worsening of both the underlying disease and the quality of life. Only subcutaneous insulin administration is recommended, and each patient must be trained to recognize, prevent, and correct hypoglycemia. Treatment considerations of diabetes in PD patients are presented in Table 4.

**Figure 1:**
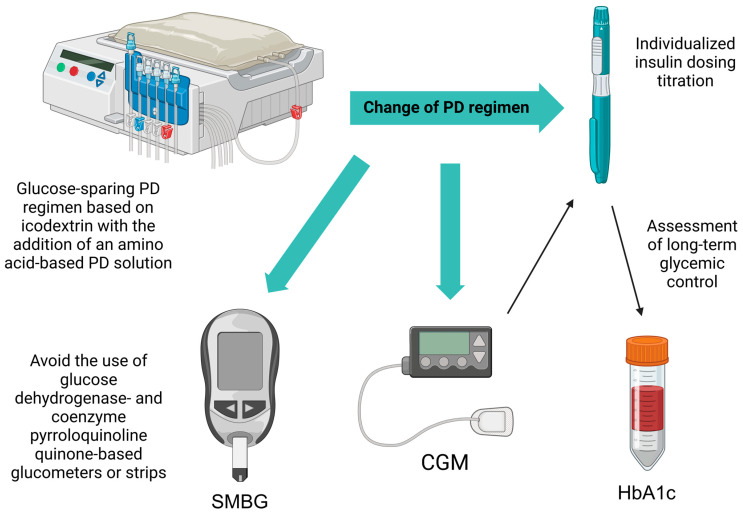
Glycoregulation in PD patients with diabetes. PD: peritoneal dialysis; CGM: continuous glucose monitoring; SMBG: self-monitoring blood glucose; HbA1c: hemoglobin A1c. The figure was created using the BioRender platform (www.biorender.com).

In patients on a PD incremental regime, a specific PD-free day insulin regime or dose change must be considered in consultation with endocrinologists. Also, each time the PD regimen is changed, either by increasing the number of changes or increasing the concentration of the glucose dialysis solution, insulin dose adjustment is necessary with hypoglycemia awareness. Since glucose monitoring is still based on capillary blood glucose measurement, three times daily premeal blood glucose levels lower than 5 mmol/L require insulin dose reduction. Patients with diabetes on PD require repeated education on avoiding hypoglycemia and objective assessment of hypoglycemia awareness [139,151]. Because of the high variability of glucose levels in PD patients with diabetes and inadequate corrections of the insulin regimen, the use of the CGM system would be of great help in preventing and resolving hypoglycemia [112]. The integrative approach to glycemic control in PD patients with diabetes on insulin therapy is presented in Figure 1.

## 9. Future Directions and Limitations

Data from prospective studies regarding the management of glycemia in people with diabetes on PD are limited. Future research should focus on assessing the clinical benefits of CGM to guide insulin management with special emphasis on time in range and long-term outcomes and how glycemic variability is associated with diabetes-related comorbidity and mortality. Additionally, the conduction of prospective trials on the use of a PD regimen consisting of more than one icodextrin exchange daily in combination with amino acid-based PD solution with just one exchange of low (1.36%) glucose dialysate solution may give answers on how to improve the treatment of PD patients with DM and whether this approach improves survival. Following the above, conducting a study evaluating the optimal use of insulin regimens in patients with DM on PD would be necessary. However, some potential limitations should be taken into account, such as the lack of availability of non-glucose PD solutions on the market in some countries. Also, in parallel with the goal of achieving the best possible glycemic control, strict monitoring of volemia control is necessary, which in some patients also implies regular administration of solutions with a higher glucose concentration.

## 10. Conclusions

In conclusion, PD patients with diabetes are at increased risk for unstable glycoregulation with high glucose variability and frequent hypoglycemic/hyperglycemic episodes that could accelerate the decline of RRF and increase the risk for atherosclerotic vascular disease. Assessment of glycoregulation by HbA1c determination is important for adjustment of anti-diabetic therapy, although HbA1c has limited accuracy. The availability of CGM gives hope for better glycemic control, but high cost limits its use. Reduced cost can be achieved through periodic CGM use, but the effectiveness of that approach in reducing glucose levels and the incidence of hypoglycemia needs to be examined in controlled studies. The use of a PD regimen with decreased content of glucose and GDP, either as a combination of icodextrin and low glucose concentration PD solution or a combination of icodextrin, amino acid-based PD solution, and low glucose concentration PD solution, achieves multiple goals such as better glycemic control, correction of lipid abnormalities, maintenance of RRF and preservation of peritoneal membrane. This strategy should be implemented immediately in nephrology centers.

## Figures and Tables

**Table 1:** Beneficial metabolic effects of icodextrin in comparison with glucose solutions.

	Glucose Solutions	Icodextrin	References
GDP	↑	↓	[20,21]
Insulin resistance	↑	↓	[64]
Triglycerides	↑	↓	[65,66,69]
LDL	↑	↓	[69,70]
VLDL	↑	↓	[65,66]
HDL cholesterol	↓	↑	[67,69,70,73]

GDP: glucose degradation products; LDL: low-density lipoprotein; VLDL: very-low-density lipoprotein; HDL: high-density lipoprotein. ↑—increase, ↓—decrease.

**Table 2:** Key findings of CGM use in PD patients with diabetes mellitus.

Study	Year	Number of Participants with DM	CGM Device	Main Results of the Study
Oei et al. [125]	2016	3	Dexcom G4	The use of CGM may provide more meaningful estimates of glucose control.
Schwing et al. [126]	2004	7	Medtronic Minimed	Increase in sensor glucose after PD exchange.
Lee et al. [119]	2013	25	Medtronic Minimed	Increase in sensor glucose within 60 min of refilling glucose-based PD exchange, and reduced sensor glucose during icodextrin dwell.
Okada et al. [121]	2015	20	Medtronic Gold	33% time glucose level was above 10 mmol/L in patients with mean HbA1c of 5.9%
Qayyum et al. [127]	2016	60	Dexcom G4	The occurrence of sensor-detected hypoglycemia even in patients with HbA1c > 9%
Marshall et al. [128]	2003	8	Medtronic Minimed	Introduction of icodextrin exchange in PD regime was associated with lower glycemic variability.
Skubala et al. [23]	2010	16	Medtronic Minimed	Mean sensor glucose and mean changes in sensor glucose were significantly greater in high transporters in comparison with high-average transporters.
Ng et al. [129]	2023	30	Medtronic Guardian Sensor 3	Satisfactory performance of CGM sensor (paired readings against gold standard Yellow Spring Instruments for venous glucose measurement).

CGM—continuous glucose monitoring; PD—peritoneal dialysis.

**Table 3:** Summary of practical aspects of CGM use in PD patients with DM.

Practice Points
1. It is efficacious in minimizing high glucose variability associated with PD exchanges (glucose influx during PD dwell).
2. It allows more accurate insulin dose titrations.
3. Small studies demonstrated the benefits of CGM use in PD patients, but results from large studies are missing.
4. For high-risk patients more than 50% per day, the target range for blood glucose level should be between 3.9 and 10.0 mmol/L, and less than 1% of the time, blood glucose value may be below 3.9 mmol/L (less than 15 min per day) and less than 50% per day it may be above the 10.0 mmol/L.
5. CGM is not widely available and has a high cost.

CGM—continuous glucose monitoring; PD—peritoneal dialysis.

**Table 4:** Approach to the treatment of diabetes in PD patients.

	Options	Additional Remarks
Oral antidiabetic drugs	Dipeptidyl peptidase-4 inhibitors (sitagliptin 25 mg daily, or linagliptin 5 mg daily) Pioglitazone (15–30 mg)	- Sulphonylureas and metformin are contraindicated - Glucagon-like peptide receptor agonists are not recommended - SGLT2i are still not approved for use in dialysis patients
Insulin therapy	A multiple-daily-injection regime with once daily long-acting (basal) analog insulin (50% of complete daily insulin dose is basal insulin and the other 50% are made up of multiple short-acting prandial/bolus premeal analog insulins) Insulin pump when indicated	- Night-time dosing of long-acting insulin analog in CAPD patients depends on the timing of PD long dwell - If high glucose values after each PD exchanges are detected, additional doses of short-acting insulin starting with 2 units can be given - For insulin pump users basal rates can be changed accordingly to changes in PD glucose load

SGLT2i: Sodium-glucose Cotransporter-2 Inhibitors; CAPD: continuous ambulatory peritoneal dialysis; PD: peritoneal dialysis.

## Supplementary Materials

The following supporting information can be downloaded at: https://www.mdpi.com/article/10.3390/life15050798/s1, Figure S1: Flow chart of the search strategy.

## Abbreviations

The following abbreviations are used in this manuscript:

DM	Diabetes mellitus
ESRD	End-stage renal disease
PD	Peritoneal dialysis
HbA1c	Hemoglobin A1c
GDP	Glucose degradation products
AGEs	Advanced glycation end products
CGM	Continuous glucose monitoring
T2D	Type 2 diabetes
HD	Hemodialysis
BMI	Body mass index
RRF	Residual renal function
NODM	New-onset diabetes mellitus
MS	Metabolic syndrome
IR	Insulin resistance
Ty/G	Triglyceride/glucose index
RAGE	Receptor for advanced glycation end products
T1D	Type 1 diabetes
CKD	Chronic kidney disease
VLDL	Very-low-density lipoprotein
HDL	High-density lipoprotein
Apo	Apolipoprotein
LDL	Low-density lipoprotein
IGT	Impaired glucose tolerance
IFG	Impaired fasting glucose
OGTT	oral glucose tolerance test
ADA	American Diabetes Association
FG	Fasting glucose
GA	Glycated albumin
HOMA	Homeostasis model assessment
CRP	C-reactive protein
CAPD	Continuous ambulatory peritoneal dialysis
PSTR	Peritoneal solute transfer rate
KDIGO	Kidney-Disease Improving Global Outcomes
ESAs	Erythropoiesis-stimulating agents
ISPD	International Society for Peritoneal Dialysis
GDH	*Glucose dehydrogenase*
GDH-PQQ	*Glucose dehydrogenase* with pyrroloquinolinequinone
GOx	*Glucose-oxidase*
TIR	Target range
TAR	Time above range
TBR	Time below range
GLP-1 RA	Glucagon-like peptide receptor agonists
DPP-4	Dipeptidyl peptidase-4
SGLT2i	Sodium-glucose Cotransporter-2 Inhibitors
GLUT	Glucose transporter
TGF	*Transforming growth factor*
VEGF	*Vascular endothelial growth factor*
CTGF	*Connective tissue growth factor*
HIF	*Hypoxia-inducible factor*
SMBG	Self-monitoring blood glucose
