# Supplementary figures and images for: Glycemic Control in Patients with Diabetes on Peritoneal Dialysis: From Glucose Sparing Approach to Glucose Monitoring
Source: Life (Basel). 2025 May 17;15(5):798. doi: 10.3390/life15050798 (PMC12113379; doi:10.3390/life15050798)

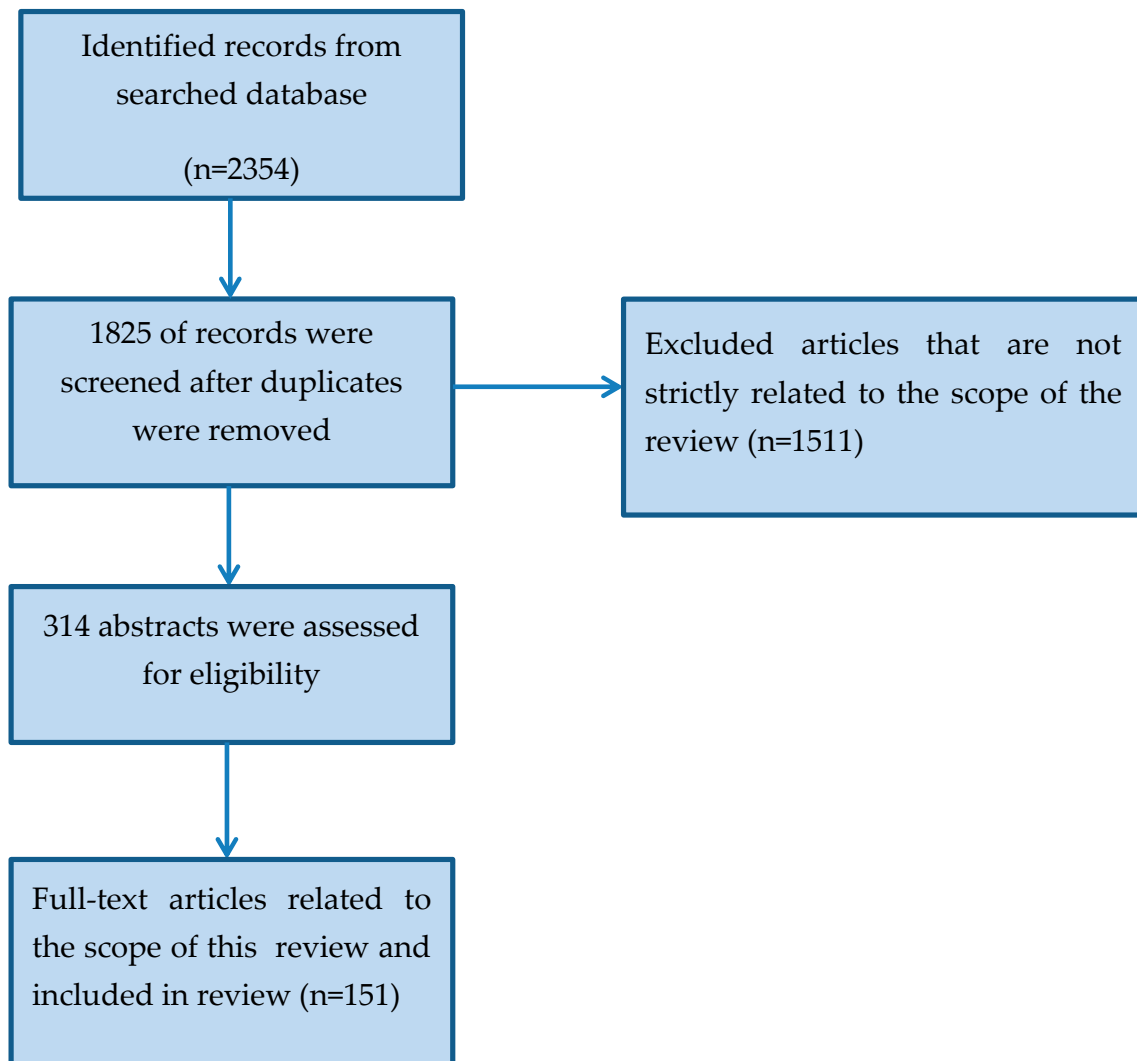

Supplemental Figure S1. Flow chart of the search strategy

Supplement: Supplementary file 1 [file life-15-00798-s001.zip › life-3604953-supplementary.pdf]
